# A Man with Juvenile Nasopharyngeal Angiofibroma, Vestibular Schwannoma, Cleft Lip and Cleft Palate, and Various Nevi: Case Report and Review

**DOI:** 10.7759/cureus.3304

**Published:** 2018-09-14

**Authors:** Boya Abudu, Philip R Cohen

**Affiliations:** 1 School of Medicine, University of California San Diego, La Jolla, USA; 2 Dermatology, San Diego Family Dermatology, San Diego, USA

**Keywords:** acoustic, angiofibroma, cleft, juvenile, lip, nasopharyngeal, neuroma, palate, schwannoma, vestibular

## Abstract

Juvenile nasopharyngeal angiofibroma is a vascular tumor that typically occurs in men. Vestibular schwannoma (acoustic neuroma) is a tumor affecting the vestibulocochlear nerve. A 38-year-old man with various pigmented lesions and history of juvenile nasopharyngeal angiofibroma, vestibular schwannoma, and cleft lip and cleft palate is described. Characteristics of patients with coexisting juvenile nasopharyngeal angiofibroma and vestibular schwannoma are summarized. A search, using PubMed, was performed for the following terms: acoustic, angiofibroma, blue, cleft, combined, dysplastic, juvenile, lip, nasopharyngeal, neuroma, nevus, palate, schwannoma, and vestibular. The relevant papers were obtained and their references were reviewed. Only one man with coincident juvenile nasopharyngeal angiofibroma and vestibular schwannoma has previously been described. The juvenile nasopharyngeal angiofibromas were both right-sided and diagnosed when the patients were 13 years old and 20 years old. Our patient’s vestibular schwannoma was ipsilateral with his juvenile nasopharyngeal angiofibroma and incidentally diagnosed on a magnetic resonance imaging (MRI) scan that was performed to monitor for recurrent juvenile nasopharyngeal angiofibroma when he was in his 30s. The other man’s vestibular schwannoma and juvenile nasopharyngeal angiofibroma were diagnosed concurrently, when he was 20 years old. The observation of a patient with cleft lip and cleft palate, various melanocytic nevi, juvenile nasopharyngeal angiofibroma, and vestibular schwannoma is unique. Although the appearance of juvenile nasopharyngeal angiofibroma and vestibular schwannoma may be coincidental, the occurrence of these tumors in the same individual may suggest an association with regards to their pathogenesis.

## Introduction

Juvenile nasopharyngeal angiofibroma is a rare vascular tumor arising in the lateral nasopharynx; it usually occurs in adolescent men [1,2]. Vestibular schwannoma (also known as acoustic neuroma) is an intracranial tumor derived from Schwann cells of the vestibulocochlear nerve [3]. We describe a man who has juvenile nasopharyngeal angiofibroma and vestibular schwannoma in addition to cleft lip, cleft palate, and various melanocytic lesions.

## Case presentation

A 38-year-old German man presented with darkly pigmented skin lesions on the back and chest. His past medical history was significant for right-sided cleft lip and cleft palate (which were repaired in early childhood), juvenile nasopharyngeal angiofibroma, and vestibular schwannoma. His family history was remarkable for melanoma in a paternal uncle.

He was diagnosed with a right-sided juvenile nasopharyngeal angiofibroma 25 years ago; the tumor had multiple recurrences and required several surgeries. His most recent recurrence, 18 years ago, involved a large infratemporal fossa approach to the right middle ear that resulted in obliteration of the external acoustic canal and right nasal septum deviation. He has since been without recurrence. However, there is stable decreased sensation in the region innervated by the second and third divisions of the right fifth cranial nerve.

A right-sided vestibular schwannoma was incidentally diagnosed six years ago during routine magnetic resonance imaging (MRI) scan monitoring for recurrent juvenile nasopharyngeal angiofibroma. Subsequently, the schwannoma has grown to 0.9 cm; it has remained stable for the last six years. Therefore, management of the vestibular schwannoma has only consisted of close observation.

**Figure 1:**
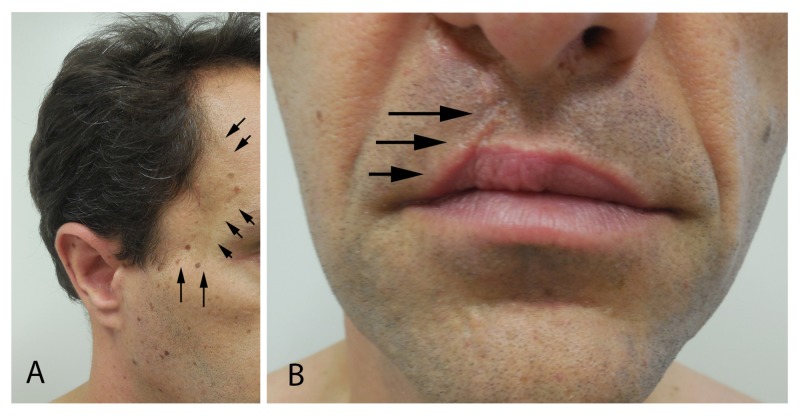
Juvenile nasopharyngeal angiofibroma and cleft lip and cleft palate. A 38-year-old man has atrophy of the right temporal area (arrows) resulting from the surgical treatment of his recurrent juvenile nasopharyngeal angiofibroma (A). He also has a healed scar (arrows) on the right side of his upper lip following repair of his cleft lip and cleft palate (B).

Cutaneous examination showed atrophy of the right temporal area secondary to surgical interventions for juvenile nasopharyngeal angiofibroma; in addition, the repair site of the cleft lip and cleft palate is noted on the right side of his upper lip (Figure 1).

**Figure 2:**
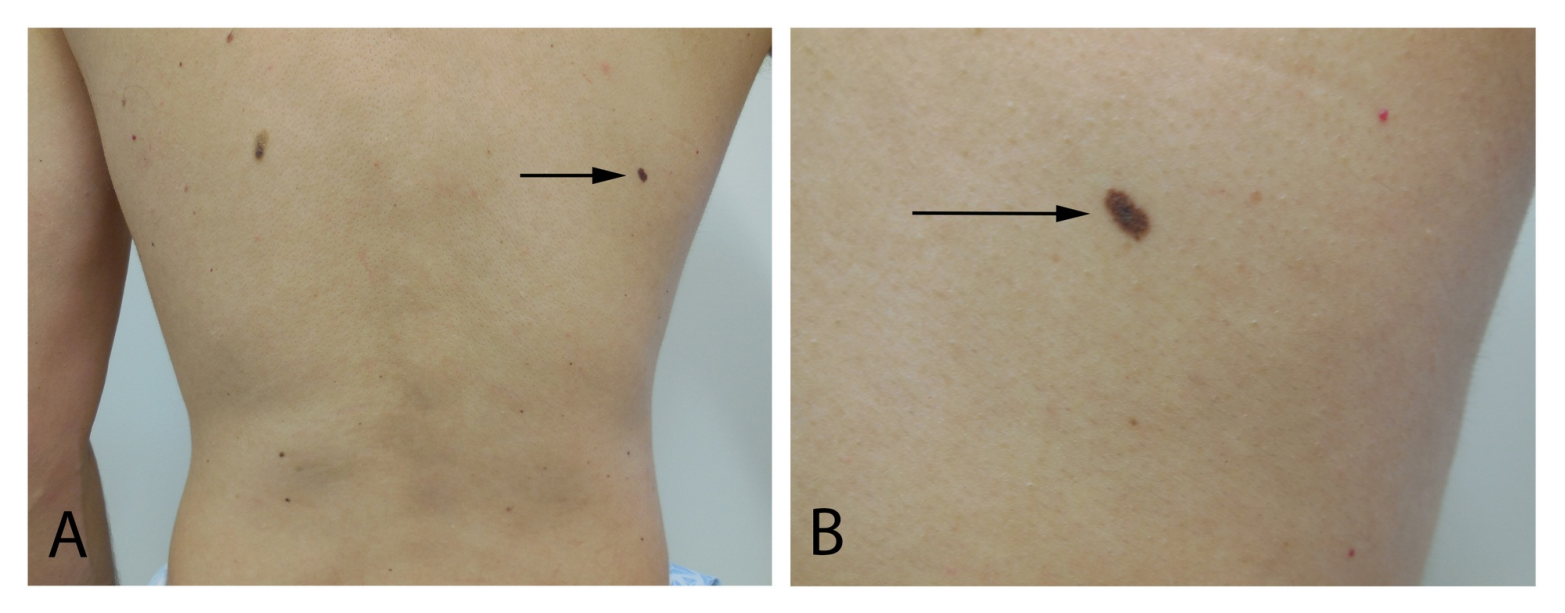
Compound dysplastic nevus with mild atypia on the right mid-back. Distant (A) and closer (B) views of an oval dark brown patch (arrow) on the right mid-back. Microscopic examination of the shave biopsy, which completely removed the lesion, demonstrated a compound dysplastic nevus with mild atypia.

**Figure 3:**
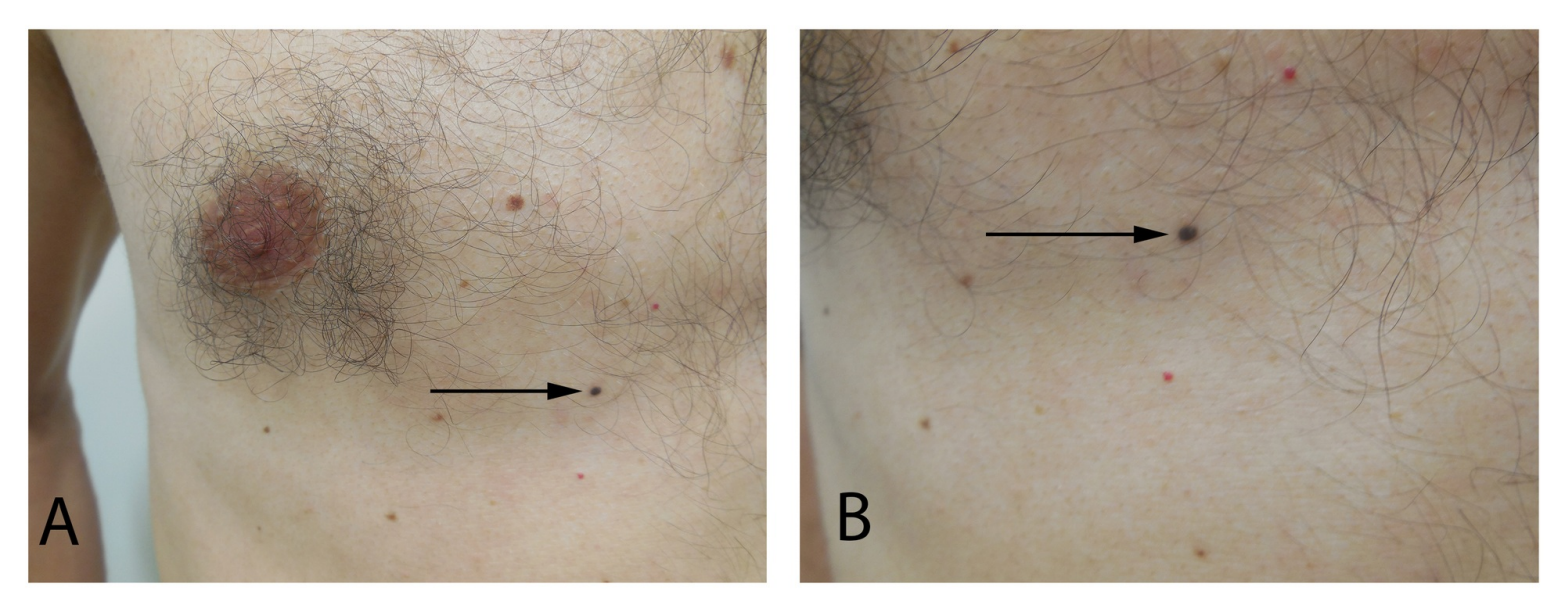
Combined (blue and intradermal) nevus of the right mid-chest. Distant (A) and closer (B) views of a small black macule (arrow) on the right mid-chest. Microscopic examination of the shave biopsy, which completely removed the lesion, demonstrated a combined nevus composed of a blue nevus and an intradermal nevus.

An 8 x 3 mm oval dark brown patch is present on the right mid-back and a 2 x 2 mm black macule is noted on the right mid-chest (Figures 2, 3). Biopsy of both skin lesions was performed.

Microscopic examination of the back lesion showed a compound dysplastic nevus with mild atypia. The chest lesion showed a combined (blue and intradermal) nevus. Both pigmented lesions had been completely removed at the time of biopsy.

## Discussion

Cleft lip and cleft palate, juvenile nasopharyngeal angiofibroma, and vestibular schwannoma may each occur as individual entities. However, our patient has all three of these conditions in addition to various pigmented lesions.

Cleft lip, with or without cleft palate, is the most common craniofacial deformity in the newborn [4]. This malformation presents with unilateral, bilateral, or central indentation of the upper lip and may extend to the roof of the mouth. Thirty percent of cleft lip and 50% of cleft palates are associated with genetic syndromes [5]. Although the etiology of spontaneous oral clefting is unknown, it is thought that a combination of genetic factors and the prenatal environment predispose to the development of cleft lip and cleft palate by inhibiting palatal, medial nasal, and maxillary process fusion. Maternal smoking, alcohol, folate deficiency, and the use of certain medications (particularly anticonvulsants) have been associated with cleft lip and cleft palate [6].

Surgical correction is curative for cleft lip and cleft palate. Repair may be initiated as early as two to three months of age for cleft lip and six to 12 months of age for cleft palate [7]. To the best of our knowledge, an association of cleft lip and cleft palate with juvenile nasopharyngeal angiofibroma has not been described.

Juvenile nasopharyngeal angiofibroma is a benign vascular tumor accounting for 0.05% of all head and neck tumors [8]. The tumor originates lateral to the sphenopalatine foramen of the nasopharynx. Juvenile nasopharyngeal angiofibroma almost exclusively occurs in prepubertal and adolescent men; however, there are a few reports of juvenile nasopharyngeal angiofibroma in either women or older adults [9-11]. The etiology of juvenile nasopharyngeal angiofibroma is unknown; however, upregulation of hormonal receptors and vascular endothelial growth factor are thought to play a role in its pathogenesis [12].

Nasal obstruction and severe, unilateral epistaxis are the most common presenting symptoms of juvenile nasopharyngeal angiofibroma. Surgery is the treatment of choice. The use of endoscopic approaches to remove the tumor has replaced external interventions [13,14].

Vestibular schwannoma is a benign intracranial tumor of vestibulocochlear nerve Schwann cells. This neoplasm was historically known as an “acoustic neuroma”; however, this is a misnomer. Although vestibular schwannomas occur spontaneously, researchers have found an association with radiation exposure during childhood [15].

Bilateral vestibular schwannomas are the hallmark of neurofibromatosis type two, an autosomal dominant disorder due to a mutation in the *NF2* gene responsible for merlin, which is a tumor suppressor. The diagnosis of neurofibromatosis type two is based on the revised Manchester criteria: bilateral vestibular schwannomas or unilateral schwannomas with established risk factors, including a first degree relative with neurofibromatosis type two or specific intracranial tumors [16]. Our patient did not fulfill the criteria for neurofibromatosis type two; he only had a unilateral vestibular schwannoma without any other risk factors.

Presenting symptoms of vestibular schwannoma often include either asymmetric hearing loss or other focal neurological deficits or both. The mainstays of therapy are either observation, surgery, or radiation therapy. Similar to our patient, when observation is decided, MRI scan or computerized axial tomography scan is repeated every six to 12 months to monitor tumor growth.

The occurrence of juvenile nasopharyngeal angiofibroma and vestibular schwannoma has been reported in another patient (Table 1) [17]. The man was 20 years old when he presented with epistaxis. A right-sided juvenile nasopharyngeal angiofibroma, as well as an incidental left-sided vestibular schwannoma, was discovered on MRI scan. No other symptoms were present.

**Table 1:** Coincident juvenile nasopharyngeal angiofibroma and vestibular schwannoma. C: Case; CR: Current report; JNA: Juvenile nasopharyngeal angiofibroma; mm: millimeter; Ref: Reference; VS: Vestibular schwannoma.

C	Sex	JNA age	JNA side	VS age	VS side, size	Ref.
1	Man	20 years	Right	20 years	Left, 10 mm	[17]
2	Man	13 years	Right	Early 30s	Right, 9 mm	CR

Our patient was asymptomatic when he was incidentally found to have a right-sided vestibular schwannoma on annual MRI scan for juvenile nasopharyngeal angiofibroma monitoring. In addition, he had a history of cleft lip and cleft palate as well as several pigmented lesions including a dysplastic nevus and a combined nevus. Although the multiple concurrent conditions may be coincidental, their expression in a single individual may provide insight into a related pathogenesis for their development. Currently, there are no consistent chromosomal or embryological abnormalities that connect juvenile nasopharyngeal angiofibroma, vestibular schwannoma, cleft lip and cleft palate, and pigmented nevi.

## Conclusions

Cleft lip and cleft palate, juvenile nasopharyngeal angiofibroma, and vestibular schwannoma are uncommon conditions that usually occur in isolation. In addition to our patient, juvenile nasopharyngeal angiofibroma and vestibular schwannoma have only been reported in one other man. To the best of our knowledge, the additional features of cleft lip and cleft palate and various pigmented lesions with juvenile nasopharyngeal angiofibroma and vestibular schwannoma have not previously been described. The concurrent presence of these conditions may be coincidental; however, their coexistence may provide insight into an associated pathogenesis.
